# Maternal deaths caused by eclampsia in Brazil: a descriptive study from 2000 to 2021

**DOI:** 10.61622/rbgo/2024rbgo65

**Published:** 2024-07-26

**Authors:** Victor Hugo Palhares Flávio-Reis, Yago Marcos Pessoa-Gonçalves, Alan de Castro Barbosa, Chamberttan Souza Desidério, Wellington Francisco Rodrigues, Carlo José Freire Oliveira

**Affiliations:** 1 Universidade Federal do Triângulo Mineiro Uberaba MG Brazil Universidade Federal do Triângulo Mineiro, Uberaba, MG, Brazil.

**Keywords:** Eclampsia, Preeclampsia, Obstetrics, Epidemiology, Brazil

## Abstract

**Objective:**

Eclampsia is a hypertensive disorder that occurs during pregnancy and can lead to death. The literature has gaps by not providing comprehensive data on the epidemiology of the disease, restricting analysis to limited temporal intervals and geographical locations. This study aims to characterize the epidemiological profile of women who died from eclampsia in Brazil from 2000 to 2021.

**Methods:**

The maternal mortality data were obtained from the *Sistema de Informações sobre Mortalidade*, with the following variables of interest selected: “Federative Unit,” “Year,” “Age Range,” “Race/Color,” and “Education Level.” The collection of the number of live births for data normalization was conducted in the Sistema de Informações sobre Nascidos Vivos. Statistical analyses were performed using GraphPad Prism, calculating odds ratio for variables and fixing number of deaths per 100,000 live births for calculating maternal mortality ratio (MMR).

**Results:**

There was a downward trend in maternal mortality rate during the study period. Maranhão stood out as the federative unit with the highest MMR (17 deaths per 100.000 live births). Mothers aged between 40 and 49 years (OR = 3.55, CI: 3.11–4.05) presents higher MMR. Additionally, black women showed the highest MMR (OR = 4.67, CI: 4.18–5.22), as well as mothers with no educational background (OR = 5.83, CI: 4.82–7.06).

**Conclusion:**

The epidemiological profile studied is predominantly composed of mothers with little or no formal education, self-declared as Black, residing in needy states and with advanced aged. These data are useful for formulating public policies aimed at combating the issue.

## Introduction

Preeclampsia is characterized by elevated blood pressure occurring typically after 20 weeks of gestation accompanied by proteinuria, or in absence but combined with hematological complications, renal insufficiency, impaired liver function, neurological symptoms, or uteroplacental dysfunction PE.^(1,2)^ Despite the mechanisms involved in hypertensive disorders during pregnancy not being fully elucidated, the pathophysiology of eclampsia is extremely complex and multifactorial, involving immunological and cytological factors.^(3)^ It is understood that the intricate process of preeclampsia may be facilitated by a combination of abnormal placentation and ischemia.^(4)^ This condition leads to the release of pro-inflammatory and anti-angiogenic proteins into maternal circulation, ultimately resulting in endothelial dysfunction, which manifests as the clinical syndrome observed in patients with preeclampsia.^(5)^ Over the years, the prevalence of preeclampsia has been increasing in Brazil, possibly as a consequence of the adoption of new diagnostic methods.^(6)^ Defined as the convulsive manifestation of preeclampsia and categorized as the most severe form of hypertensive disorders, eclampsia exhibits high morbidity and mortality for both the mother and the fetus.^(7,8)^ Indeed, eclampsia contributes to more than 50,000 annual deaths worldwide.^(9)^

Despite advancements in eclampsia prevention and treatment, notably through the administration of Magnesium Sulphate, which has demonstrated a substantial reduction in the risk of eclampsia and maternal mortality, the incidence of this disease remains a significant public health concern.^(10-12)^ In developing nations, eclampsia occurs at a rate of 50 to 151 per 10,000 deliveries. In contrast, developed countries report 1.6 to 10 per 10,000 deliveries.^(13)^

The current epidemiological literature on maternal deaths resulting from eclampsia in Brazil often analyses specific geographic regions and shorter temporal periods. In this context, the present study aims to conduct a longer period temporal analysis of maternal mortality caused by eclampsia in Brazil from 2000 to 2021, encompassing the entirety of the Brazilian territory and including the analysis of sociodemographic characteristics as predictors of the odds of dying from eclampsia. The updating of epidemiological data related to this condition assumes fundamental importance for mediating health indicators, thereby contributing to informed decision-making in public health management.

## Methods

This study was conducted in accordance with the guidelines of the STROBE protocol for observational studies and have an epidemiological nature, being an ecological time-series study.^(14)^ The designated time frame for this analysis, from January 2000 to December 2021, was chosen to enhance the understanding of fluctuations in variables related to deaths caused by the disease over the 21^st^ century. The year 2021 was chosen as the endpoint of the analysis because the available databases provided information up to that year.

Data regarding the number of maternal deaths due to eclampsia in Brazil between 2000 and 2021 were collected from the *Sistema de Informações sobre Mortalidade* (SIM) utilizing the corresponding International Classification of Diseases 10 code O15 for eclampsia.^(15)^ The study population consisted of women aged 10 to 59 years who died due to eclampsia between 2000 and 2021, and the selected variables of interest included “Year,” “Federation Unit,” “Age group,” “Race/ethnicity,” “Education level,” and “Marital status”.

For conducting descriptive statistical analyses and calculating the Maternal Mortality Ratio (MMR), expressed as the ratio between the number of maternal deaths and the number of live births, Microsoft Excel software, version 2309 from 2019, was employed. The data were tabulated, and the calculation was established by fixing the number of deaths per 100,000 live births, a data obtained from the *Sistema de Informações sobre Nascidos Vivos* (SINASC).^(16)^

For the calculation of the relative risk and odds ratio for all variables, the analyzed variables were considered as the exposure factor, with eclampsia-related death as the outcome. Since neither the *Departamento de Informática do Sistema Único de Saúde* (DATASUS) nor the *Instituto Brasileiro de Geografia e Estatística* (IBGE) provides the number of pregnant women’s who did not die from eclampsia, the number of live births was used instead. They are obtained from the SINASC, for the relative risk calculation. The chi-square test with Yates’ correction was performed, in addition to the Fisher’s exact test using 0.05 (5%) significance level.

Graphs were created using GraphPad Prism, version 8.0.0, and QGIS, version 3.34.0, being the data used for the development of graphical representations obtained from the IBGE website. D’Agostino & Pearson and Shapiro-Wilk tests were employed to assess the normality of the selected variables. Additionally, Pearson and Spearman tests were used to investigate the correlation between variables using 0.05 (5%) significance level.

## Results

**Figure 1:**
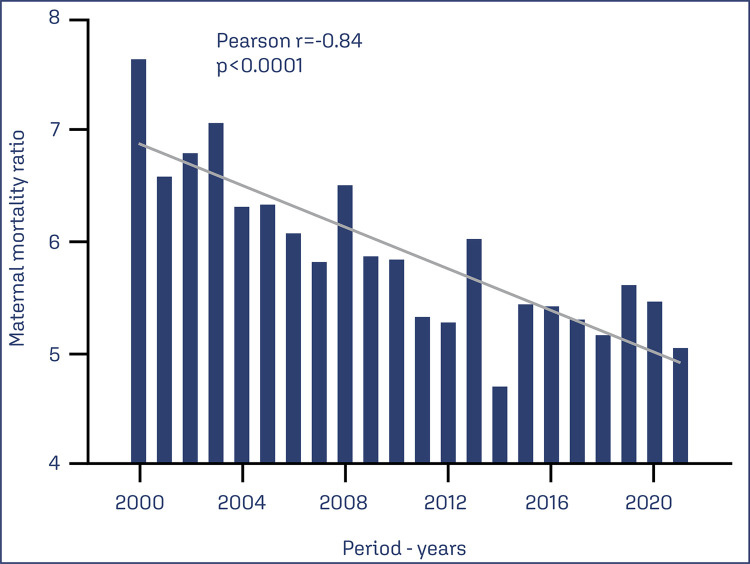
Maternal Mortality Ratio (MMR) per year caused by eclampsia between 2000 and 2021. Data were collected from the Sistema de Informações sobre Mortalidade (SIM) and the *Sistema de Informações sobre Nascidos Vivos* (SINASC), with MMR standardized per 100,000 live births

During the period under analysis, a total of 3823 maternal deaths attributed to eclampsia were documented. Temporal analysis of the distribution of eclampsia-related deaths across the selected years unveiled a decrease in the MMR per year, accompanied by a significant negative temporal correlation (r=-0.84, p<0.0001), as illustrated in figure 1. The MMR averaged 5.88 maternal deaths per 100,000 live births per year, with a standard deviation (SD) of 0.70. The year 2000 had the highest MMR, reaching 7.6 deaths per eclampsia for every 100,000 live births, while the year 2014 had the lowest, with a rate of 4.6 deaths per eclampsia for every 100,000 live births.

**Figure 2:**
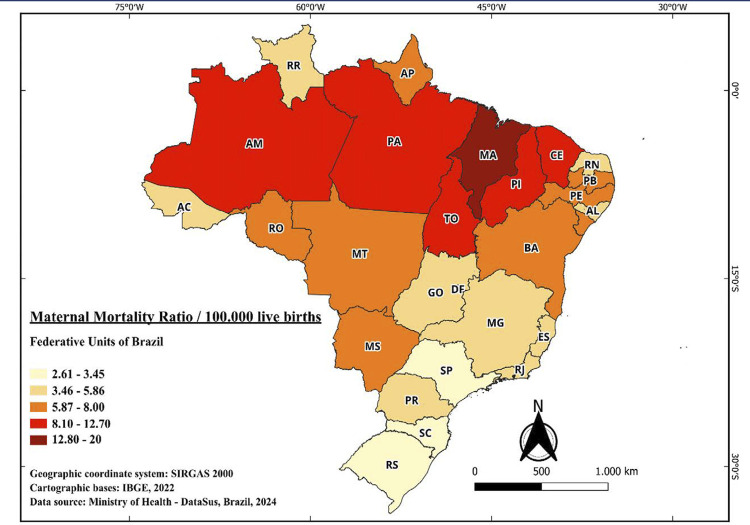
Maternal Mortality Ratio (MMR) due to eclampsia by Brazilian Federal Unit between 2000 to 2021. Data were collected from the Sistema de Informações sobre Mortalidade (SIM) and the Sistema de Informações sobre Nascidos Vivos (SINASC), standardizing the MMR per 100.000 live births

Among the 27 individual federative units examined, an average MMR of 6.5 deaths per 100,000 live births was observed over the period, with a SD of 3.2. The Federative Units that exhibited highest MMR for the entire period were Maranhão, Piauí, Pará, and Tocantins, with 17.03, 12.67, 11.97, and 10.47 deaths per 100,000 live births, respectively. Conversely, the states with lower MMR were the Distrito Federal, Santa Catarina, and Rio Grande do Sul, with 3.18, 3.12, and 2.61, respectively, as illustrated in figure 2.

**Figure 3:**
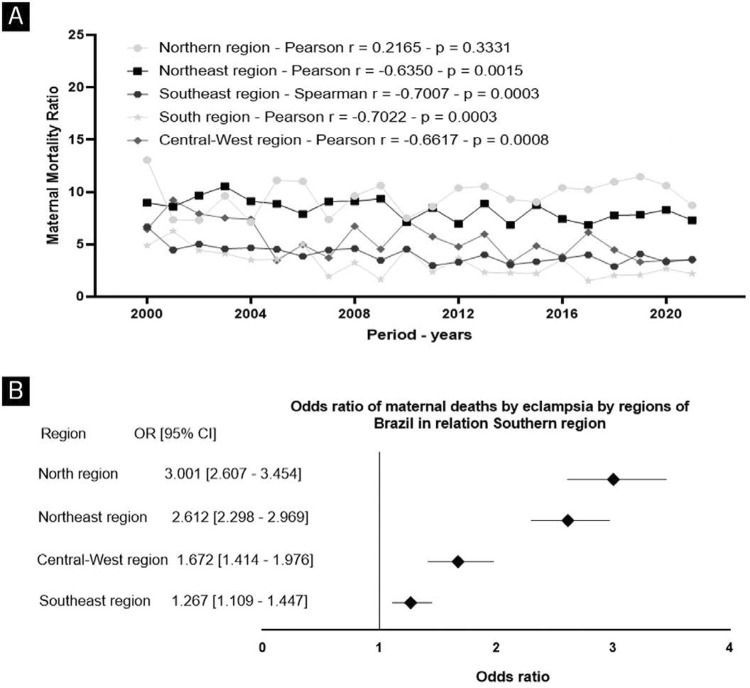
(A) Temporal correlations and Maternal Mortality Ratio due to eclampsia across regions in Brazil over time (2000-2021). Pearson’s and Spearman’s tests were employed for correlation calculations. *(B) Odds ratio of maternal deaths by eclampsia between Southern (region with lowest maternal death) to other Brazilians regions*

Analyzing the temporal evolution of the MMR due to eclampsia across different regions of Brazil (Figure 3A), revealed that only the Northern (AC, AM, RR, AP, PA, TO, and RO) region did not show a temporally and statistically significant correlation, while all other regions exhibit a decreasing trend. When comparing averages among regions over the period, the Southern (MMR average of 3.18, SD of 1.28), composed by RS, SC and PR states, and Southeastern (MMR average of 4.04, SD of 0.85), composed by SP, MG, RJ and ES states, regions had significantly lower rates during the period. The Northeast (r = -0.63), composed by BA, MA, PI, AL, PE, PB, RN, CE and SE states, Southeast (r = -0.70), South (r = -0.70), and Central-West (r = -0.66), composed by MT, MS, GO and DF states, regions showed a significant negative temporal correlation (p < 0.05), whereas the Northern region (r = 0.21) did not exhibit a significant temporal correlation (p > 0.05). Furthermore, the odds ratio of a woman dying from eclampsia in the Northern region are three times higher than in the Southern region (Figure 3B).

Regarding the age group (Table 1), it was observed that mothers aged between 50 and 59 years exhibited the highest MMR due to eclampsia during the analyzed period (35.54 deaths per 100,000 live births). On the other hand, mothers aged between 20 and 29 years showed the lowest MMR (approximately 4.58 maternal deaths per 100,000 live births). This observation reveals a significant disparity in MMR, being the relative risk 7.7 times higher in the first group compared to the second, highlighting the substantial influence of age on maternal mortality associated with eclampsia.

**Table 1:** Maternal Mortality Ratio per age group in the Federative Units of Brazil

Federative Units	10–14 years	15–19 years	20–29 years	30–39 years	40–49 years	50–59 years
Rondônia	16.55	5.31	5.69	8.53	0	0
Acre	0	7.64	3.84	2.71	13.30	0
Amazonas	15.06	8.03	5.72	13.7	25.27	0
Roraima	0	3.6	3.21	10.05	0	0
Pará	23.7	12.12	9.06	17.67	35.99	279.33
Amapá	41.64	5.07	4.7	11.69	59.54	0
Tocantins	0	11.31	7.05	15.09	72.65	0
Maranhão	42.31	17.71	12.59	23.86	68.25	330.03
Piauí	23.71	7.53	11	21.12	24.54	0
Ceará	0	7.42	6.86	10.31	27.31	0
Rio Grande do Norte	0	4.22	4.17	5.95	12.09	0
Paraíba	7.92	3.87	4.35	11.66	13.27	0
Pernambuco	6.3	4.46	5.39	8.34	20.61	0
Alagoas	18.07	6.24	3.42	7.98	16.40	0
Sergipe	0	4.55	6.85	11.69	14.91	0
Bahia	11.74	4.87	4.13	10.66	22.73	0
Minas Gerais	2.97	3.94	3.3	5.24	8.07	0
Espírito Santo	0	3.9	2.92	4.54	7.39	0
Rio de Janeiro	5.35	6.36	4.55	7.03	14.97	0
São Paulo	1.46	2.9	2.49	4.66	9.93	0
Paraná	3.85	4.03	2.57	4.7	17.65	0
Santa Catarina	0	2.34	2.68	3.77	9.14	0
Rio Grande do Sul	0	1.64	2.19	3.25	7.44	0
Mato Grosso do Sul	9.07	6.89	6.21	7.41	0	0
Mato Grosso	15.59	5	4.6	8.74	28.99	0
Goiás	6.07	4.46	4.9	6.81	17.34	0
Distrito Federal	0	0.76	3.07	3.44	13.41	0
Total	10.27	5.86	4.59	7.41	16.32	35.55
Odds ratio	2.24	1.27	-	1.61	3.55	7.74
Confidence interval (95%)	1.72–2.90	1.16–1.39	-	1.49–1.74	3.11–4.05	1.93–31.01

Data shows a higher mortality at extremes of age (extreme ages of menacme). MMR is the ration between the number of maternal deaths by eclampsia and the number of 100,000 live births. The Odds Ratio represents the maternal deaths by eclampsia by age group in relation to mothers aged between 20–29 years.

**Figure 4:**
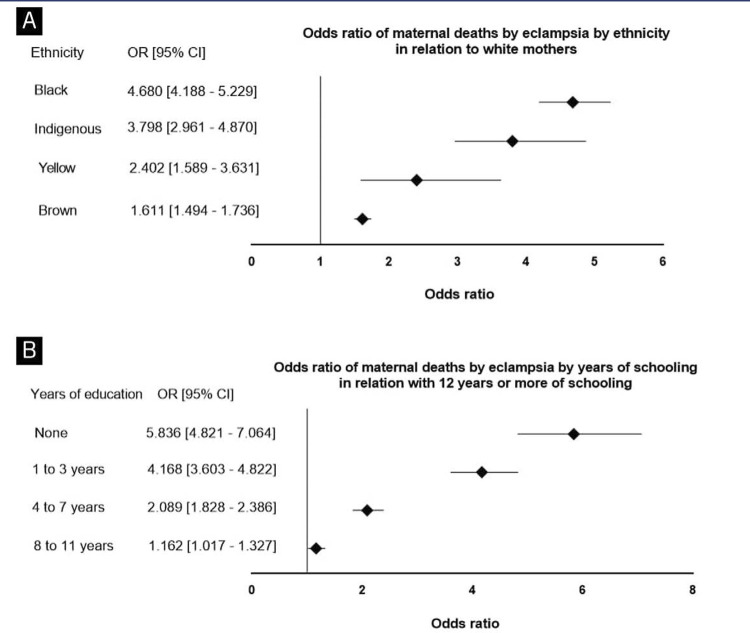
(A) Odds ratio for ethnicity. (B) Odds ratio for years of education. Maternal Mortality Ratio by mother’s level of education. Data of mother’s level of education were collected from the *Sistema de Informações sobre Mortalidade* (SIM) and the *Sistema de Informações sobre Nascidos Vivos* (SINASC), standardizing the Maternal Mortality Ratio per 100,000 live births for each group

A noteworthy finding during the correlation between the variables “Age Group” and “Federal Unit” is the identification that the state of Maranhão showed the highest MMR across various age groups, except for the age group of 40 to 49 years, where Tocantins emerged with the highest MMR. Examining the MMR based on the variable of ethnicity, it was evident that the “white” ethnicity recorded 3.93 deaths per eclampsia for every 100,000 live births, while the “brown” ethnicity had a rate of 6.34. Additionally, the “yellow” ethnicity showed an MMR of 9.46, followed by the “indigenous” ethnicity with 14.95, and the “black” ethnicity with the highest rate, reaching 18.43. Correlating this variable with the mother’s age group, we observed that among mothers who self-identified as black, those in the age group of 40 to 49 years were the most affected. Calculating the odds ratio (Figure 4A), it was observed that the risk of a black woman dying from eclampsia is 4.68 times higher than that of a white woman (OR = 4.679 95%, CI: 4.188 - 5.229). Furthermore, exploring the MMR based on the mother’s level of education revealed a notable reduction proportional to an increase in the number of years of education. The MMR significantly decreased, starting at 15.72 for mothers with no years of education and reaching the lowest point of 2.69 for those with 12 or more years of formal education. While calculating the Odds Ratio (Figure 4B), it was observed that the risk of a woman dying from eclampsia due to having no years of schooling is 5.83 times higher when compared to those with 12 years or more of education (OR= 5.83; CI: 4.82– 7.06).

## Discussion

Eclampsia remains one of the predominant health conditions associated with maternal deaths in Brazil.^(17)^ The data collected and analyzed reveal a negative temporal correlation, indicating a decrease in maternal mortality from eclampsia over the years. However, despite this decrease, potential social determinants contributing to higher maternal mortality from eclampsia in specific groups were identified, such as black women and mothers with no educational background.

Regarding the decrease in the MMR due to eclampsia over the 22-year temporal analysis, it is plausible to attribute this decline to strategies implemented by the Ministry of Health within the scope of prenatal care. Among these strategies, the introduction of programs such as the *Programa de Humanização Pré-Natal*^(18)^ and *Rede Cegonha*^(19)^ stands out, which incorporated standardized models of care. It is worth mentioning, for example, that this programs adopted prenatal practices based on widely recognized and recommended models in developed nations, such as attention to the care process during pregnancy and childbirth, the coordination of care points in a network, and obstetric regulation during delivery, technical qualification of primary care teams and in maternity care, improvement of the healthcare environment (Primary Health Care Units and maternity wards), expansion of services and professionals, to encourage the practice of physiological childbirth and the humanization of childbirth and delivery.^(19)^

The federative units considered in the study as having the highest MMR (Maranhão, Piauí, Pará, and Tocantins) stood out. This fact is explained by the states’ poor rates of prenatal care, attributed to specific health factors intrinsic to each state.^(20)^ Among these factors are the centralization of health centers, resulting in longer distances for mothers to travel to the hospital and consequently a delay in prenatal care,^(21)^ refusals of care due to overcrowding at the hospital sought, as well as the provision of prenatal care in public hospital facilities.^(22)^

Moreover, the observation that the likelihood of death due to eclampsia is threefold higher in the northern region compared to the southern region aligns with the current scientific literature.^(23)^ This phenomenon can be explained by the lower frequency of prenatal care visits among women from the Northern region, as evidenced by the literature.^(24)^

Although the literature indicates a higher incidence of hypertensive disorders during pregnancy, including eclampsia, among women over 40 years old,^(25)^ this study also demonstrates a high MMR in women aged above 40 years. This finding aligns with well-established risk factors associated with pregnancy at advanced ages, such as gestational diabetes, placenta previa, caesarean delivery, prolonged hospitalization and preterm prelabor rupture of membranes.^(26)^

As mentioned earlier, significant disparities in the MMR were observed among different ethnicities and maternal education levels. The alarming number of deaths among black women aligns with data already documented in the literature, highlighting the main causes of this scenario: the unfavorable socioeconomic conditions among black women, resulting in poorer prenatal and childbirth care, the lower proportion of black women with a companion during childbirth, with over 50% of them not having a companion due to healthcare service disapproval, the longer waiting times for assistance during childbirth for Black women, the higher proportion of normal deliveries among Black women compared to White women, the less information received about normal childbirth and the childbirth location by Black women, the lower participation in educational activities in healthcare services by Black women, and the lower proportion of Black women who received care at the first healthcare service sought compared to white women. Indeed, it is important to emphasize that there is no biological reason that puts black women at a higher risk of maternal death. Rather, socioeconomic conditions are the determinants of this outcome.^(27,28)^

A study conducted with women in Sweden, Norway, Denmark, Finland, and Iceland, in various contexts,^(29)^ emphasized that a lower formal level of education is associated with a 20% higher probability of developing hypertensive disorders during pregnancy. Similarly, this study demonstrates low education as an important factor in the higher MMR. This can be attributed to the lower understanding of pregnant women regarding the importance and necessity of professional care and self-care during pregnancy,^(30)^ as well as potential difficulties in accessing healthcare services.^(31)^

The data in the present study, especially those referring to the final years encompassing the COVID-19 pandemic, did not show significant correlations with increased maternal mortality due to eclampsia. However, studies have shown that pregnant women with COVID-19 may have a higher risk of developing pre-eclampsia.^(32,33)^ Considering that prenatal care during the pandemic was reduced, it cannot be ruled out that there was possible underreporting of cases, influencing the data.^(34)^

## Conclusion

This work stands out by presenting a more comprehensive analysis, incorporating a diversity of variables and covering a more extended temporal period, potentially serving as a foundation for public policy intervention aimed at improving the presented epidemiological scenario. The epidemiological panorama of maternal deaths exclusively attributed to eclampsia in Brazil, spanning from 2000 to 2021, shows a decreasing trend over the years, predominantly encompasses Black women, aged over 50 years, widowed marital status, and with virtually nonexistent levels of education. Thus, this study contributes to the expansion of knowledge in the literature, as the scarcity of epidemiological studies exclusively focused on maternal deaths attributed to eclampsia in Brazil for the selected period is notable.
